# Patterns of Vaping Behavior and Perceived Health Risks Among Young Adults: A Cross-Sectional Study

**DOI:** 10.1192/j.eurpsy.2026.10851

**Published:** 2026-07-31

**Authors:** O. Martin-Santiago, B. Arribas-Simon, M. Calvo-Valcarcel, P. Martinez-Gimeno, M. Rios-Vaquero

**Affiliations:** Hospital Clinico Universitario de Valladolid, Valladolid, Spain

## Abstract

**Introduction:**

Vaping has gained popularity since its introduction in 2005, particularly among adolescents and young adults, due to its appealing flavors and perceived lower health risks compared to traditional smoking. Despite its widespread use, emerging evidence suggests that vaping may pose significant health risks, including respiratory and cardiovascular damage, and potential neurological effects.

**Objectives:**

This study aimed to explore the demographic and behavioral characteristics of individuals who vape, assess the perceived health risks associated with vaping, and examine the relationship between vaping and conventional tobacco use.

**Methods:**

A cross-sectional exploratory design was employed. A non-probabilistic convenience sample of 230 individuals aged 18–40 was recruited via social media. Participants completed a questionnaire assessing vaping habits, substance types used, motivations, perceived risks, and tobacco use history.

**Results:**

Of the 230 participants, 97 (42.2%) reported having vaped at least once. Among them, 66.0% no longer vape, while 5.2% vape daily, 6.2% occasionally, and 22.7% sporadically. The primary motivations for initiating vaping were curiosity (60.8%) and social influence (15.5%), with fewer citing smoking cessation (11.3%) or stress reduction (2.1%).

A significant proportion (62.9%) perceived vaping as equally harmful as smoking, and 80.4% reported quitting due to health concerns. A positive correlation was found between risk perception and intention to quit (r = .372, p < .001).

Regarding substance use, 59.8% used nicotine-containing liquids, while 35.1% used nicotine-free variants. Only 5 participants reported using cannabis-derived substances. A significant association was found between nicotine vaping and tobacco use (χ^2^(1, N = 92) = 8.21, p = .004), with 77.8% of smokers using nicotine vapes compared to 48.9% of non-smokers (see **Image 1**).

**Image 1:**
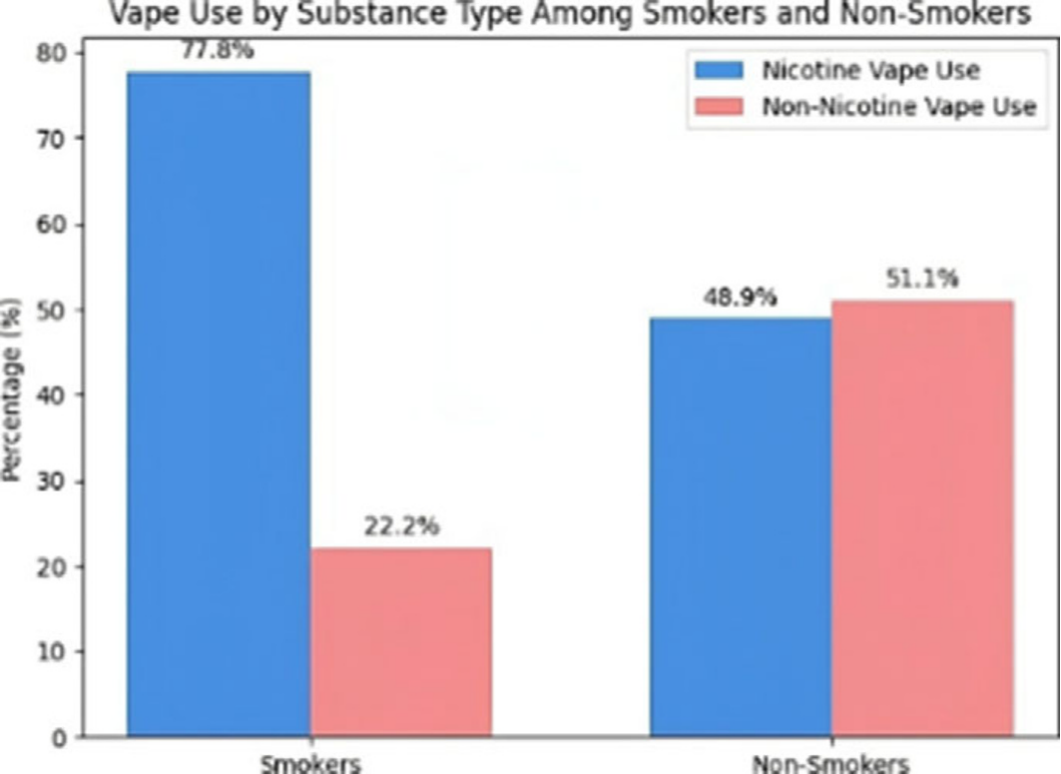

**Conclusions:**

The findings indicate that while most individuals discontinue vaping, a notable minority continue the practice. Curiosity and peer influence are dominant initiation factors, contrasting with previous studies that emphasize stress relief and smoking cessation as primary motives. The high prevalence of nicotine use among non-smokers raises concerns about potential nicotine dependence in previously non-smoking populations.

Compared to earlier research suggesting vaping may aid in reducing cigarette consumption, this study highlights a more complex reality where vaping does not consistently serve as a cessation tool. Moreover, the perception of vaping as harmful appears to be a protective factor, suggesting that public health campaigns should emphasize risk awareness to reduce usage.

**Disclosure of Interest:**

None Declared

